# Real-Time PCR for Molecular Detection of Zoonotic and Non-Zoonotic *Giardia* spp. in Wild Rodents

**DOI:** 10.3390/microorganisms9081610

**Published:** 2021-07-28

**Authors:** Christian Klotz, Elke Radam, Sebastian Rausch, Petra Gosten-Heinrich, Toni Aebischer

**Affiliations:** 1 Unit 16 Mycotic and Parasitic Agents and Mycobacteria, Department of Infectious Diseases, Robert Koch-Institute, 13353 Berlin, Germany; radame@rki.de (E.R.); gosten-heinrichp@rki.de (P.G.-H.); aebischera@rki.de (T.A.); 2 Centre for Infection Medicine, Institute of Immunology, Freie Universität Berlin, 14163 Berlin, Germany; sebastian.rausch@fu-berlin.de

**Keywords:** *Giardia* spp., wild rodents, molecular detection, qPCR

## Abstract

Giardiasis in humans is a gastrointestinal disease transmitted by the potentially zoonotic *Giardia duodenalis* genotypes (assemblages) A and B. Small wild rodents such as mice and voles are discussed as potential reservoirs for *G. duodenalis* but are predominantly populated by the two rodent species *Giardia microti* and *Giardia muris*. Currently, the detection of zoonotic and non-zoonotic *Giardia* species and genotypes in these animals relies on cumbersome PCR and sequencing approaches of genetic marker genes. This hampers the risk assessment of potential zoonotic *Giardia* transmissions by these animals. Here, we provide a workflow based on newly developed real-time PCR schemes targeting the small ribosomal RNA multi-copy gene locus to distinguish *G. muris*, *G. microti* and *G. duodenalis* infections. For the identification of potentially zoonotic *G. duodenalis* assemblage types A and B, an established protocol targeting the single-copy gene *4E1-HP* was used. The assays were specific for the distinct *Giardia* species or genotypes and revealed an analytical sensitivity of approximately one or below genome equivalent for the multi-copy gene and of about 10 genome equivalents for the single-copy gene. Retesting a biobank of small rodent samples confirmed the specificity. It further identified the underlying *Giardia* species in four out of 11 samples that could not be typed before by PCR and sequencing. The newly developed workflow has the potential to facilitate the detection of potentially zoonotic and non-zoonotic *Giardia* species in wild rodents.

## 1. Introduction

*Giardia* spp. are flagellated protozoan parasites of vertebrates and a frequent cause of gastrointestinal disease in wild and domestic animals and humans [1]. *Giardia duodenalis* is the most widespread species in mammals and subdivided into eight genetic subgroups (assemblages A-H) [1]. These subgroups show distinct host ranges: Assemblages A and B are found in humans and a wide range of other mammals, including rodents, and are considered potentially zoonotic [2]; assemblages C/D are found in canids; assemblage E in hoofed animals; assemblage F in cats; assemblage G in rodents (in particular rats) and assemblage H in pinnipeds [1]. Small rodents can furthermore be infected with the distinct species *Giardia muris* and *Giardia microti* [1,3,4]. For both *G. microti* and, in particular, for *G. muris*, distinct morphological characteristics in comparison to *G. duodenalis* have been described [5].

The zoonotic *G. duodenalis* assemblages A and B are genetically very distinct, and each group can be further subclassified based on genetic information determined by multi-locus sequence typing (MLST) [6]. Within these subgroups, assemblage AI forms a pan-global, almost clonal subpopulation that is found in a wide range of vertebrates, including humans, whereas assemblage AII seems very genetically diverse and is almost exclusively found in humans [1]. The subclassification of assemblage B is less well-defined—in particular, when classical MLST procedures are used. Additionally, assemblage B has a higher degree of allelic sequence heterogeneity (ASH) in the tetraploid genome that further hampers isolate typing [7].

Rodents are frequently parasitized by *Giardia* spp., and the potential zoonotic risk has been highlighted by several studies [8,9,10,11,12,13]. For example, in North American wildlife, the prevalence of *Giardia*-positive beavers (*Castor canadensis*) has been estimated at 13–30%, and zoonotic transmission has been implicated by several studies [14,15,16]. A recent molecular characterization of the genomic sequences of historic samples of water, animals and humans from Canada gave further evidence that beavers can be infected with zoonotic *G. duodenalis* assemblages A and B [16]. Another rodent species that has been implicated with zoonotic transmissions is the pet chinchilla *Chinchilla lanigera*, where mainly *G. duodenalis* assemblage B-type infections have been recognized [10,17,18].

The roles of zoonotic transmission by smaller rodents commonly found in natural and human habitats, such as mice, voles and rats, are not well-resolved. The few studies providing molecular typing data revealed that these genera are mainly infected by *G. muris*, *G. microti* and/or *G. duodenalis* assemblage G, respectively [4,19,20,21,22,23]. However, experimental infections in mice and gerbils with zoonotic assemblages are possible [24,25,26,27], and in some rare cases, these were also described in nature in these animals [4,28,29]. 

Studies reporting the *Giardia* prevalence in wild rodents often lack species information or genotyping data, so that the zoonotic risk cannot be estimated from those studies. One likely reason for the lacking data is that *Giardia* species determination and assemblage typing mainly relays on a cumbersome test principle, including amplification by the (nested) PCR and sequencing of genetic marker genes, such as small subunit rRNA gene (SSU), triosephosphate isomerase (TPI), glutamate dehydrogenase (GDH) and beta giardin (BG) [1,2,3,4,6,21,30,31,32,33,34]. However, these assays are not equally reliable for all species at all marker genes, and genomic information is not readily available for all species to elaborate more adequate procedures. Currently, the only available reliable genomic marker to determine all *Giardia* species is the SSU locus.

The aim of the study was to provide a new workflow based on the available and newly developed real-time PCR protocols to determine the *G. muris*, *G. microti* and zoonotic *G. duodenalis* assemblage types in rodent samples. These protocols will help in future studies to better determine the potential risk for zoonotic *Giardia* transmission from wild rodents.

## 2. Materials and Methods 

### 2.1. Sample Material and Reference Sequences

Trophozoites of *G. duodenalis* assemblages A (isolate WB6, ATCC 50803) and B (isolate GS, ATCC 50581) were derived from axenic in vitro cultures using standard culture procedures [35]. Cysts of *G. muris* (Thompson isolate) were commercially obtained (Waterborne Inc, New Orleans, LA, USA). Fecal samples of *G. muris* and *G. duodenalis*-infected mice were obtained from experimental laboratory infections done in a different context. These procedures were approved by the local authorities, the Landesamt für Gesundheit und Soziales (G0277-17, G0207/19).

DNA samples of wild rodents are described elsewhere [4]. DNA of *Balamuthia mandrillaris*, *Toxoplasma gondii* and *Leishmania donovani* were previously extracted from routine in vitro cultures and available from archived materials. DNA of *Entamoeba histolytica* axenic cultures was a kind gift from Prof. Iris Bruchhaus (Bernhard Nocht Institute, Hamburg, Germany). 

The following reference sequences (GenBank accession numbers) of the SSU gene locus were used to design *Giardia* species-specific oligonucleotides: *G. muris* (X65063 and AF113895), *G. microti* (AF006676 and AF006676), *G. duodenalis* assemblage A (M54878 and AF199446), *G. duodenalis* assemblage B (AF199447 and AF3898), *G. duodenalis* assemblage C (AF199449), *G. duodenalis* assemblage D (AF199443), *G. duodenalis* assemblage E (AF199448), *G. duodenalis* assemblage F (AF199444) and *G. duodenalis* assemblage G (AF199450). These references were supplemented with the sequence information of *G. muris* and *G. microti* derived from a screen in wild rodents (KY114167-KY114486) [4]. The aligned sequence fragment comprised approximately 250 bp, as determined by a *Giardia*-specific nested PCR and sequencing approach, as described earlier [4,30,36]. Currently, this sequence fragment is the one with the largest sequence collection of different *Giardia* isolates in the public databases, including all three mammalian *Giardia* species and assemblage types. In addition, due to its comparably high copy number in the genome, the ribosomal gene loci has been widely used for genotyping and phylogenetic approaches, as it promises supposedly higher PCR detection sensitivity [37]. The design of primers and probes to distinguish *G. muris*, *G. microti* and *G. duodenalis* was done using the Geneious software tool (Biomatters, Auckland, New Zealand).

For determination of the *G. duodenalis* assemblage types A and B, we used a previously described protocol that detects the single-copy target gene 4E1-HP [38].

### 2.2. DNA Extraction and Real-Time PCR

DNA extraction of samples was done with the QIAamp Fast DNA Stool Mini Kit (Qiagen, Hilden, Germany) according to the manufacturer’s protocols. Real-time PCR assays were standardized and performed using single PCR approach as the multiplexing reduced assay performance (data not shown). Conditions were as follows using the 2X Maxima Probe/ROX qPCR Master Mix (Thermo Fisher, Schwerte, Germany) in a 25 µL PCR reaction: 12.5 µL reaction buffer, 1 µL of each forward and reverse primer (from 3.3-µM stock), 0.5 µL of probe (from 3.3-µM stock), 1–5 µL DNA template and add water for the final volume. Reactions were started at 95 °C for 10 min, followed by 45 cycles of 95 °C denaturation for 15 s, 60 °C annealing for 30 s and 72 °C extension for 30 s. Real-time PCR and analysis was performed using a CFX Maestro PCR machine and the respective software tools (both from Bio-Rad, Feldkirchen, Germany). Sequences of the primers and probes used in the study are presented in Table 1. All real-time PCR assays for specificity and analytical sensitivity were repeated in 3 separate experiments for each run in duplicates. For specificity testing, 2µL of 50 pg/µL DNA was used in the PCR. For sensitivity testing, a serial dilution of DNA was performed, ranging from 50 pg/µL to 0.5 fg/µL, and 2µL of diluted DNA was used in the PCR assay. Primary samples were tested once in duplicates, and the results were presented as mean cq values. Samples were only considered PCR-positive when both duplicates gave an amplification signal. The inhibition of PCR was controlled using an internal amplification control and was not found to be an issue [4]. Data of the sensitivity testing were analyzed using Prism (GraphPad, San Diego, CA, USA), and plots were presented as mean cq values (*n* = 3) with 95% confidence intervals. 

## 3. Results

### 3.1. Development of Real Time PCR Workflow to Distinguish G. muris, G. microti and G. duodenalis

**Figure 1:**
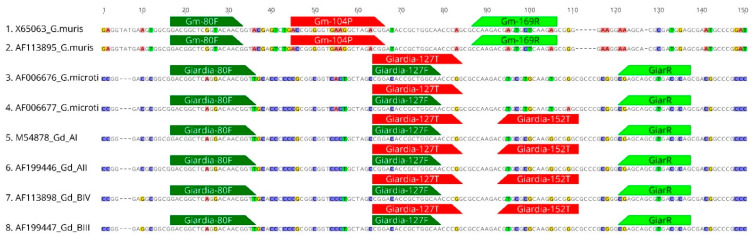
Alignment of the partial SSU gene locus of selected *Giardia* reference sequences illustrating the position of the primers and probes of the newly developed real-time PCR approaches. Forward primers in dark green, reverse primers in light green and probes in red.

An approximately 250 bp *SSU* genome fragment (see Methods for details) was used for the primer design. At this locus, a well-established and robust real-time PCR to diagnose *G. duodenalis* in humans has been previously described [39]. Furthermore, significant differences at this locus sequence between *G. muris* on the one hand and *G. duodenalis* and *G. microti* on the other hand allowed for the design of a *G. muris*-specific primer and probe combination (Figure 1 and Table 1). In contrast, *G. duodenalis* and *G. microti* sequences showed high overall similarities, so that no *G. microti-*specific real time PCR could be designed (Figure 1). The data analysis included 104 previously identified *G. microti* sequences ([4], not shown), which revealed a high variability at the 3’-end of the sequence fragment, and this region was therefore not reliable for primer design.

Instead, an alternative approach was chosen to distinguish *G. microti* from *G. duodenalis*: First, sequences were evaluated for highly conserved regions shared by both *G. duodenalis* and *G. microti*, and a PCR was designed to distinguish these two species from *G. muris*. Secondly, the sequences were evaluated for regions that distinguish *G. duodenalis* from *G. microti* to design a *G. duodenalis*-specific PCR probe (Table 1 and Figure 1). In combination, these two PCR approaches allow the distinction between *G. microti*-positive and *G. duodenalis*-negative samples.

**Figure 2:**
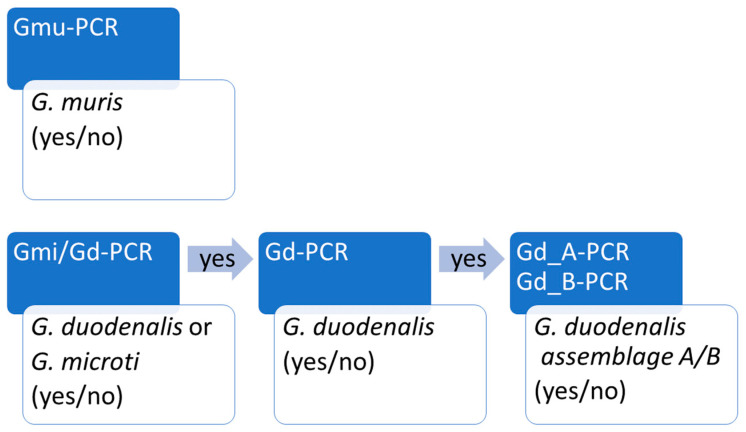
Workflow of the real-time PCR approach used to determine *G. muris*, *G. microti* and zoonotic *G duodenalis* in rodent samples.

To further identify and distinguish zoonotic assemblages A and B, we used a real-time PCR assay developed by Vanni et al. 2012 [40] and modified by Pijnacker et al. 2016 [38]. Using these five PCR approaches allowed us to identify all three *Giardia* species and zoonotic assemblage types (see the workflow in Figure 2).

### 3.2. Specificity of Real-Time PCR Assays

After the primer and probe selection, we tested the specificity of the single PCR assays. We therefore used DNA from various sources, including fecal DNA samples of infected rodents and the DNA of axenic cultures or purified cysts where available. We confirmed the expected specificity of all real-time PCRs, as shown in Table 2. 

### 3.3. Analytical Sensitivity of Real-Time PCR Assays

**Figure 3:**
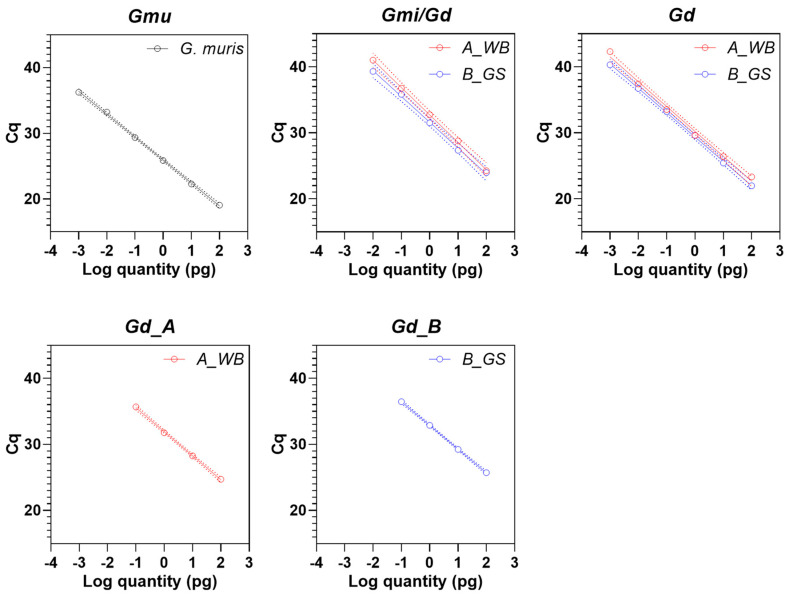
Analytical sensitivity of the real-time PCR assays used in the study. The *G. muris*-specific PCR (*Gmu*) was done with DNA from purified cysts. *G. microti/G. duodenalis* PCR (*Gmi/Gd*), *G. duodenalis*-specific PCR (*Gd*), *G. duodenalis* assem-blage A-specific PCR (*Gd_A*) and *G. duodenalis* assemblage B-specific PCR (*Gd_B*) was done using the DNA of axenic trophozoite cultures of assemblage A isolate WB (*A_WB*) and of assemblage B isolate GS (*B_GS*).

To test the analytical sensitivity of the PCR assays, we used extracted DNA from axenically cultured *G. duodenalis* trophozoites or from purified *G. muris* cysts (Figure 3). We were not able to test the sensitivity of *G. microti*, as no adequate material was readily available for this *Giardia* species.

The assay designed for the detection of *G. muris* (*Gmu* PCR) indicated a high sensitivity reporting 1 fg DNA with a cq value of 36.3 ± 0.65. This equals approximately 0.1 genome equivalents for this species (estimated genome size of *G. muris* is 9.8 MB [41]). 

The DNA of *G. duodenalis* assemblages A and B were equally well-detected in the *G. duodenalis/G. microti* (*Gmi/Gd* PCR) approach, with a sensitivity of 10 fg (equals about 0.7 genome equivalents, cq value 41.0 ± 1.52 for assemblage A and 39.3 ± 0.95 for assemblage B) and, in the *G. duodenalis*-only (*Gd* PCR) approach, with a sensitivity of about 1 fg DNA (42.3 ± 1.39 cq values for assemblage A and 40.3 ± 1.27 cq values for assemblage B), which equals about 0.07 genome equivalents (estimated genome size of *G. duodenalis* assemblage A is 12.6 MB [42]), respectively. The sensitivity of the former PCR was reproducibly lower and did not reliably detect the 1 fg DNA sample dilution.

Sensitivity of the *G. duodenalis* assemblage A- and B-specific PCR was also similar for both assemblage types, but only about 100 fg DNA (cq value 35.7 ± 0.29 for assemblage A and 36.5 ± 0.26 for assemblage B) or approximately 7 genome equivalents per PCR reaction were detected. The lower sensitivity was expected due to the single-copy target versus multi-copy target in the former PCRs. 

### 3.4. Reanalysis of Known Giardia Positive Wild Rodent Samples Confirms Applicability of PCR Workflow for Detection of G. muris, G. microti and Zoonotic G. duodenalis Assemblages

To test the applicability of the new PCR approach, we reanalyzed 38 *Giardia*-positive samples from a previous project [4] (Table 3). These samples consisted of samples tested positive for *G. muris*, *G. microti* or *G. duodenalis*, respectively, as previously determined by nested PCR and the sequencing of a fragment at the SSU locus [4]. We also included samples for which the *Giardia* species could not be determined in the previous study. 

All nine *G. muris* samples were confirmed by our new *G. muris*-specific real-time PCR. In addition, the new method revealed an underlying coinfection by a putatively non-zoonotic genotype of *G. duodenalis* in one sample (Isolate 301, Table 3, negative in A- and B-specific PCR). As highlighted above, the lack of a direct *G. microti*-specific PCR (as we were not able to appropriately design one) does not allow to formally exclude an additional underlying *G. microti* infection. However, in this case, a lower cq value in the *Gd/Gmi* combi-PCR compared to the *Gd*-only PCR would be expected, which was not the case.

Of the 14 *G. microti* samples, 12 were confirmed by our new PCR workflow. Unexpectedly, two samples exhibited a signal in the *G. muris*-specific PCR with relatively low cq values of 31, but no signal was recorded in the *Gd/Gmi*-PCR. We can only speculate on the reason and favor the possibility of an underlying double infection with a lower *G. microti* load that may have been amplified in the previous nested PCR approach. The nested PCR tends to amplify *G. microti* (and *G. duodenalis*) sequences with higher sensitivity than that of *G. muris* (see also below).

Two of the four *G. duodenalis* samples were also confirmed by our assay; however, two samples were not confirmed, likely due to lower sensitivity of the real-time assay compared to the nested PCR approach used in the previous study (see, also, Discussion below). One sample was further confirmed as a zoonotic assemblage type, whereas the other sample (exhibiting a high cq value in the two *Gd* PCR assays) was not confirmed. Again, the reason is most likely lower sensitivity of the assemblage-specific PCR that targets single-copy genes in comparison to the PCR at the multi-copy SSU locus.

Four of the 11 samples that could not be typed in our previous study displayed a positive signal in the *G. muris* real-time PCR, which indicates a possible underestimation of the *G. muris* prevalence in the previous work [4]. One sample also showed a signal in the *Gd*-specific PCR; however, as the *Gmi/Gd* PCR was negative, we do not consider this result as reliable.

## 4. Discussion

Here, we provided a real-time PCR workflow for the detection of *G. muris*, *G. microti* and *G. duodenalis* infections in small rodents, including the detection of zoonotic *G. duodenalis* assemblage types A and B. The workflow provided an improved time-to-results procedure for the analysis of rodent fecal samples to determine the *Giardia* species and for an assessment of the potential zoonotic risk.

Two rodent species are well-described to harbor the potential zoonotic *G. duodenalis* assemblages A and B, and the zoonotic transmission has been described [16,43]. One is the North American beaver, and a recent study provided the first in-depth molecular analysis based on whole-genome sequencing of historical outbreak samples from various water, beaver and human sources and clearly highlighted the presence of zoonotic assemblages in the water and beaver samples [16]. The sequenced samples were mostly derived from in vivo propagation in gerbils and by in vitro cultivation. This could lead to a potential bias in the propagated assemblages, and beavers may also carry other assemblages or *Giardia* species. Pet chinchillas harbor zoonotic *G. duodenalis* (mostly assemblage B) and non-zoonotic assemblages C, D and E [10,17,18,43,44,45]. 

For ubiquitous small mammals such as mice and voles, the potential relevance of zoonotic transmission is largely unknown. Most of the larger screens for zoonotic pathogens that included *Giardia* in their analyses were based on the microscopic detection of cysts and rarely included molecular tools to determine the *Giardia* species or assemblage types. The potential relevance for zoonotic transmissions was nevertheless often discussed [13,46]. Here, we provided a new approach that could be used in future studies to facilitate the determination of *Giardia* species and may help to assess the potential relevance of rodent species for zoonotic transmissions of *Giardia* infections. 

The results presented here indicated that our workflow was sensitive and robust for such purposes. However, our method also had clear limitations. The most prominent limitation was that *G. microti* cannot be detected directly. We based the design of the primers and probes on previously acquired sequence information at the SSU locus of 106 unique *G. microti* and 10 unique *G. muris* sequences [4]. To our knowledge, this currently reflects the largest sequence collection available for these species. For *G. microti*, the sequence analysis revealed a broad variety of genotypes even at the usually highly conserved SSU gene locus [4]. Due to the sequence variety and shortage of further sequence information, we lacked the appropriate oligonucleotides for a direct *G. microti*-specific PCR and needed to rely on PCR signals obtained with primers detecting *G. microti*/*G. duodenalis* or solely *G. duodenalis*, respectively. Notably, a previously described real-time PCR for human diagnostic purposes [39] partially detected some, but not all, *G. micoti* genotypes (data not shown), hence providing little benefit to the current study. 

The analysis revealed different sensitivities for the various PCR approaches, which was important for the interpretation of the results. However, all PCRs detected one or less genome equivalents at the SSU locus and about 10 genome equivalents at the 4E1-HP locus. This was in good concordance with previously published results using these loci [47,48]. It is unclear why *G. microti/G. duodenalis*-specific real-time PCR is less sensitive than *G. duodenalis* PCR in particular, because the same region is covered. Possibly, conformational hindrance at the gene locus may influence the binding affinity of the primer and probe. For the *G. duodenalis* assemblage-specific PCR, the lower sensitivity was likely due to the single-copy target gene used for detection. The lower sensitivity compared to real-time PCR at the SSU locus has been also described previously [38]. 

Whether the low-level detection of pathogens in real-time PCR assays, as indicated by high cq values, are indeed relevant for zoonotic transmissions should be carefully evaluated in future screening studies. For example, in our original study, an analysis of 358 *SSU* sequences derived from wild rodents revealed only five *G. duodenalis*-positive samples, and these samples were not typable at the single locus genes *TPI*, *GDH* and *BG*. Four of these samples were included in the present study and revealed either very low cq values for *G. duodenalis* qPCR or *G. duodenalis* was undetectable with the presented PCR methods. Only one sample revealed detectable amounts of *G. duodenalis* assemblage B DNA, confirming our previous typing results. Overall, this implied a very low zoonotic risk in the studied German rodent population. 

In conclusion, we provided a processive and robust workflow for the detection of all *Giardia* species populating small rodents, including the zoonotic *G. duodenalis* assemblage types. This approach could be used in future studies to estimate the possible relevance for zoonotic transmissions by these animals.

## Figures and Tables

**Table 1:** Primers used in the study.

Target Species Specificity (Abbreviation of qPCR Assay) and Oligonucleotides	Oligonucleotide Sequence	Reference
*G. muris (Gmu)*		
Gm80F	5‘-GACGGCTCGGTACAACG-3‘	This study
Gm169R	5‘-CTCTTGAGCACTCGTCTTGG-3‘	This study
Gm104P	FAM-5‘-ACCGGGGGTGAAGGCTAGACGG-3‘-BHQ1	This study
*G. microti/G. duodenalis (Gmi/Gd)*		
Giardia-80F	5‘-GACGGCTCAGGACAACGGTT-3‘	[39]
GiaR	5‘-CTGCGTCACGCTGCTCG-3‘	[32]
Giardia-127T	TexasRed-5’-CGGACACCGCTGGCAACCCGG-3’-BHQ2	This study
*G. duodenalis (Gd)*		
Giardia-127F	5‘-CGGACACCGCTGGCAA-3‘	This study
GiaR	5‘-CTGCGTCACGCTGCTCG-3‘	[32]
Giardia-152T	HEX-5‘-GCCCGCCCTTGCGCGCACG-3‘-BHQ2	This study
*G. duodenalis* Assemblage A (*Gd_A*)		
4E1-HP-Af	5’-AAAGAGATAGTTCGCGATGTC-3’	[38,40]
4E1-HP-Ar	5’-ATTAACAAACAGGGAGACGTATG-3’	[38,40]
4E1-HP-Atp	VIC-5’-aggcacacggtttacaccg-3’-BHQ1	[38]
*G. duodenalis* Assemblage B (*Gd_B*)		
4E1-HP-Bf	5’-GAAGTCATCTCTGGGGCAAG-3’	[38,40]
4E1-HP-Br	5’-GAAGTCTAGATAAACGTGTCGG-3’	[38,40]
4E1-HP-Btp	TexasRed-5’-TACACTGTTCGTATGACCACTGTCGATA-3’-BHQ2	[38]

**Table 2:** Specifics of the qPCR-assays.

Sample Species	Material for DNA Extraction	PCR-Assay				
		Gmu	Gmi/Gd	Gd	Gd_A	Gd_B
*G. muris*	Feces of lab mouse ^1^	+	−	−	−	−
*G. muris*	Purified cysts	+	−	−	−	−
*G. duodenalis* A ^2^ (WB6)	In vitro culture	−	+	+	+	−
*G. duodenalis* B (GS) ^2^	In vitro culture	−	+	+	−	+
*G. duodenalis* B (GS) ^2^	Feces of lab mouse ^1^	−	+	+	−	+
*G. microti*	Feces of wild bank vole ^1^	−	+	−	−	−
*Balamutia mandrilaris*	In vitro culture	−	−	−	−	−
*Entamoeba histolytica*	In vitro culture	−	−	−	−	−
*Toxoplasma gondii*	In vitro culture	−	−	−	−	−
*Leishmania donovani*	In vitro culture	−	−	−	−	−

^1^ Lab mice were experimentally infected with the respective *Giardia* species, and *G. microti* infection of the wild bank vole was previously confirmed by PCR and sequencing [4]. ^2^ *G. duodenalis* A (WB6): assemblage A, isolate WB6 and *G. duodenalis* B (GS): assemblage B, isolate GS.

**Table 3:** Analysis of *Giardia*-positive wild rodent samples to determine the underlying *Giardia* species. Reanalysis of selected *Giardia*-positive samples from reference [4] by the established qPCR workflow.

Rodent Species	Isolate # [4]	Previous Result [4]	qPCR-Assay (Cq-Value)				
			Gmu	Gmi/Gd	Gd	Gd_A	Gd_B
*Microtus agrestis*	118	*G. muris*	27.6				
*Apodemus agrarius*	220	*G. muris*	35.0				
*Apodemus agrarius*	243	*G. muris*	36.0				
*Apodemus agrarius*	301	*G. muris*	36.1	41.3	37.1		
*Apodemus agrarius*	311	*G. muris*	32.5				
*Myodes glareolus*	328	*G. muris*	36.8				
*Myodes glareolus*	334	*G. muris*	27.9				
*Apodemus flavicollis*	385	*G. muris*	35.5				
*Apodemus flavicollis*	511	*G. muris*	30.1				
*Microtus arvalis*	443	*G. microti*		36.9			
*Myodes glareolus*	451	*G. microti*		36.6			
*Myodes glareolus*	516	*G. microti*		34.1			
*Microtus arvalis*	603	*G. microti*		34.8			
*Microtus arvalis*	495	*G. microti*		34.3			
*Microtus arvalis*	496	*G. microti*		30.9			
*Myodes glareolus*	502	*G. microti*		36.5			
*Myodes glareolus*	508	*G. microti*		29.1			
*Apodemus flavicollis*	518	*G. microti*	31.4				
*Apodemus flavicollis*	524	*G. microti*	31.1				
*Myodes glareolus*	559	*G. microti*		32.9			
*Myodes glareolus*	561	*G. microti*		29.7			
*Microtus arvalis*	566	*G. microti*		31.9			
*Microtus arvalis*	568	*G. microti*		31.9			
*Myodes glareolus*	041	*G. duodenalis* Ass. A					
*Myodes glareolus*	056	*G. duodenalis* Ass. A		40.2	39.6		
*Apodemus sp.*	207	*G. duodenalis* Ass. A					
*Myodes glareolus*	340	*G. duodenalis* Ass. B		33.0	30.2		34.5
*Apodemus sylvaticus*	305	Unknown ^1^					
*Apodemus flavicollis*	348	Unknown ^1^			41.5		
*Apodemus flavicollis*	376	Unknown ^1^					
*Apodemus sp.*	400	Unknown ^1^					
*Apodemus flavicollis*	520	Unknown ^1^					
*Apodemus agrarius*	576	Unknown ^1^	32.2				
*Myodes glareolus*	554	Unknown ^1^					
*Myodes glareolus*	555	Unknown ^1^	31.1				
*Microtus arvalis*	567	Unknown ^1^	35.8				
*Microtus arvalis*	569	Unknown ^1^					
*Myodes glareolus*	591	Unknown ^1^	36.2				

^1^ Previous nested PCR approach at the SSU locus revealed no typable results [4].

## Data Availability

Data is contained within the article.
